# For a new Sociology of Social love

**DOI:** 10.1007/s12108-023-09572-5

**Published:** 2023-03-29

**Authors:** Nicola Montagna

**Affiliations:** Department of Criminology & Sociology, Middlesex University London, London, NW4 4BT England

**Keywords:** Social love, Care, Overabounding, Social change, Sociological thought

## Abstract

Love is a theme at the centre of all our lives, including those of sociologists and social scientists. It has been widely addressed and described in literature and poetry, extensively depicted in the pictorial arts, sung about in music. Even philosophy, from its very beginnings, has devoted beautiful and intense pages to this theme. For reasons difficult to understand, the founding fathers of our discipline have been reluctant to enter the analytical realm of love. They touched this theme, but only marginally. It is only relatively recently that more insightful and focused discussions have come from some key figures of contemporary sociology in works by Niklas Luhmann, Anthony Giddens, Ulrich Beck and Elisabeth Beck-Gernsheim, Zygmunt Bauman and, more recently, Eva Illouz that demonstrate the profoundly social nature of our most intimate feelings and convey how the transformation of love and intimacy is related to wider social changes. In this sense, this collection edited by Silvia Cataldi and Gennaro Iorio aims to fill a major gap, while fuelling the debate on social love and its implications as a transformative force in an era characterised by multiple crises. By bringing together scholars from across several countries, not only it collates the fruit of years of research, but it also launches new developments in the debate on social love and set a new research agenda.

## Introduction

Love is a theme at the centre of all our lives, including those of sociologists and social scientists. It has been widely addressed and described in literature and poetry, extensively depicted in the pictorial arts, sung about in music. Love is constantly at the centre of the thoughts and turmoil of any person, regardless of gender, race, sexual orientation or religion. Even philosophy, from its very beginnings, has devoted beautiful and intense pages to this theme. For reasons difficult to understand - given the undeniable importance of love in articulating human relationships and structuring social life - sociologists and the founding fathers of our discipline have been reluctant to enter the analytical realm of love (Iorio, 2014; Rusu, 2018). They touched this theme, but only marginally. 

Durkheim devotes only a few pages to love, all concentrated in his study on the origin of incest published in L’Année Sociologique. According to the French sociologist, the contradistinction between family love and the passionate love of the couple embodies ‘the eternal antithesis between passion and duty’ (Durkheim, 1897: 67). Family love is a moral imperative and stands under the sign of duty (Rusu, 2018). It is functional to the existence of the family since, without it, the institution would collapse. In contrast, passionate love is the outcome of ‘the movement of spontaneous private sensibilities’ (p. 61). While the former is moved by the dual imperative of duty and morality, in the latter “the man and the woman seek their own pleasure and the society they form depends exclusively, at least in principle, on their elective affinities” (Durkheim, 1897, quoted in Rusu, 2018: 5).

Neither does love take a central position in the work of Max Weber: a few pages on “brotherly love” can be found when he discusses religions promising redemption in his work on the historical sociology of religion, while George Simmel writes about love in his Fragment on Love (1984 [1904]), an unfinished essay published posthumously. In the context of his reflections on everyday life love is a sociological problem insofar as it represents a particular form of social life. It is one of the elements of relations between people and as such makes society possible. Hence its sociological relevance. Finally, among the classics, we can include the Russian American sociologist Pitirim Sorokin (1950; 1954) and his influential work on Altruistic Love^1^.

Its empirical indeterminacy, which makes it difficult to observe with sociological methods, and the perception of love as a psychological phenomenon belonging to the emotional realm, or an artistic practice to be learned, have relegated love to the margins of sociological investigation. More insightful and focused discussions have come from some key figures of contemporary sociology in works by Niklas Luhmann (1986), Anthony Giddens (1992), Ulrich Beck and Elisabeth Beck-Gernsheim (1995), Zygmunt Bauman (2003) and, more recently, Eva Illouz (2012; 2018) that demonstrate the profoundly social nature of our most intimate feelings and convey how the transformation of love and intimacy is related to wider social changes^2^.

In this sense, the collection *Social Love and the Critical Potential of People* (2022), edited by Silvia Cataldo and Gennaro Iorio (Routledge: London and New York, 346 pp.), aims to fill a major gap, while fuelling the debate on social love and its implications as a transformative force in an era characterised by multiple crises. The two scholars are well known in this field and have been studying social love for nearly two decades. They co-edited *Culture of Peace* with Vera Araújo (2016), and Gennaro Iorio has published the groundbreaking *Sociology of Love* (2014). This co-edited book, that brings together scholars from across the globe – the ideas developed in this book are the result of an ongoing discussion within the international research network of Social-One, which has already conducted numerous empirical studies and edited several publications, especially in Europe and Latin America – not only collates the fruit of years of research, but also launches new developments in the debate on social love.

### The Four Dimensions of Social love

Love is understood here as a social force with an eminently public character, capable of generating change and activating community-building and solidarity processes in contemporary societies. As Cataldi and Iorio put it (p.1) “We can define it as an action, relationship or social interaction in which subjects exceed (in giving, in receiving, in not giving or not doing, in neglecting) all their antecedents, and therefore offer more than the situation requires in order to make benefits.”

Love is not a private issue, an emotion developing within each of us and remaining within our intimate sphere. Rather it is a form of collective action capable of reimagining new futures, a transformative force with a public dimension. Two steps are necessary for a sociological understanding of social love. The first is methodological and relates to the aforementioned sociological imagination. Social love and its impact on social relations can be identified through an imaginative use of a variety of sources such as newspaper reporting, interviews, surveys, case studies, and virtual ethnography. The second is to understand how the solution to some of the contemporary challenges including collective health, armed conflicts in the international context, the environment, social justice and inequalities can find inspiration from social love. The question that this book aims to answer in its 21 interdisciplinary contributions is how social love as a critical force in the lives of individuals can rethink and address these challenges.

The book, structured in five parts, is an attempt to investigate the nature of social love, its critical power for people, and how it can have a transformative impact on social reality by identifying four key dimensions. It does so from both theoretical and empirical perspectives, providing concepts, definitions, and theoretical discussions, but also studies based on empirical research. Each of its five parts is, thus, organised into an *insight* section where theoretical issues are discussed; an *evidence* section, which includes empirical chapters reporting the results of research on love; and a *current issues* section, which focuses on contemporary processes related to the analysed dimension of love and care today.

The first part sets out the theoretical and methodological foundation of the social love. Here, the authors provide an overview of the concept of social love, they show its hermeneutical power and how it can be empirically investigated. Cataldi and Iorio start by wondering about the commonality of situations as diverse as a group of local Ukrainians who feed and offer a distraught Russian soldier a cup of tea during the Russian-Ukrainian conflict, the mother of a former motocross champion in a wheelchair after an accident who can assist her son without losing her job thanks to the many unused work permits donated by colleagues and strangers, a fisherman from Lampedusa on trial for saving 24 migrants who risked drowning in crossing the Mediterranean Sea, or the shopkeepers who give surplus food to the needy in Brazil, Argentina, and in a growing number of countries to avoid food waste and help those who need it. An initial answer is that these acts are *overabounding* in the sense that they exceed shared expectations by doing more than the situation requires. In other words, they are not duties but go beyond antecedent actions. As Cataldi and Iorio write, social love is “an action, relationship or social interaction in which subjects exceed (in giving, in receiving, in not making or not doing, in neglecting) all their antecedents, and therefore offer more than the situation requires in order to make benefits” (p.12). As an action, love is practical and therefore needs closeness and concreteness. As Cataldi and Iorio put it: “It is not enough to be sensitive to the tragedy of the other or to be moved by news on television. Love needs to be close.”

However, while *overabounding* defines the character of the examples described above, this is only one dimension of social love. There are, in fact, three other dimensions that define what social love is: *care*, *universalism*, and the *recognition* of the other. The combination and co-presence of these four dimensions, which are investigated in each part of the book, make love both a typical behaviour and an unprecedented one, analytically distinct from other forms of affection, such as, for example, attention, compassion, respect.

In her theoretical chapter Cataldi traces the history of the social thought on love, beginning with the first philosophical reflections by Plato, Aristotle, and in the New Testament. She identifies four main sociological lines of investigation. First, the classics of sociological thought, such as Simmel, Weber, and Sorokin, who conceptualised love as a social form in the public sphere attributing a purely public dimension to it. Secondly, those sociologists such as Sombart, Giddens, Bauman and Illouz, among others, who moved away from the public dimension and focused on the intimate dimension of love – and how this has changed in relation to broader social changes. Third, feminist scholars such as Hite and Jackson who have re-evaluated the social dimension of love, highlighting how it represents an ideology that has legitimised patriarchy and gender oppression. Fourthly, the contribution of critical scholars such as Boltanski and Honneth, whose formulation of the concept of social love certainly contributed that of this book. Finally, Cataldi cites the most recent interdisciplinary reflections on social love.

Once social love is conceptualised and its dimensions identified, how can it be investigated? Which methodological approaches and research tools can be used to identify its various forms in the real world? As the subtitle and numerous chapters remind us, a key methodological approach is that of the *sociological imagination*, which, to use Sari Hanafi’s words (p.90), the author of one *insight* chapter in the book, “reminds us of the complex nature of social phenomena, the importance of the agency of actors, and the logic of the gift and love.” This well-known and sometimes misused concept indicates the ability to reflect on oneself by trying to transcend the familiar habits of everyday life and look at reality from a different perspective (Mills, 1959). Starting from this point, Serena Quarta, Marco Palmieri and Giuseppe Pellegrini in their chapter “For an empirical study of social world”, argue that the research strategies employed to investigate the empirical phenomena of social love can involve both quantitative and qualitative methodologies including: observation, case study, meta- analysis of case studies, survey, and analysis of secondary data. Methodological choices will depend on the scholar’s background and the object of study. However, as the authors of this chapter highlight (p.40): “qualitative and quantitative research designs on social love have a common element: the need to nurture, at the same time, the conceptual and operational dimensions of scientific action. The first dimension addresses the conceptualization of the problem under investigation and its integration within a more general theoretical framework; the second dimension guides the data collection for composing the empirical basis of the study. The methodology of social research deals with the coexistence of these two instances, to make it possible to move from words to facts, from theoretical to observational terms, from the theory to the ground of experience.” If sociologists have been reluctant to investigate social love because of its difficulties in being operationalised, this chapter raises several issues, but also provides interesting answers.

This first part concludes with two chapters showing how the concept of social love has changed across time and space. Gennaro Iorio retraces the history of social systems, showing different forms and manifestations of the human social bond around love. Building upon the work of social theorists such as Norbert Elias (1939) and Jeremy Rifkin (2009), he argues that the human race has developed methods of care that have progressively approximated the form of social love of contemporary societies. Its increasing relevance, in the form of the progressive internalisation of social rules and the rejection of violence, was investigated in Elias’s classic *The Civilizing Process*. Like Elias and Rifkin and, I would add, along the lines of Steven Pinker’s (2011) work, Iorio links changes in structural social organisations to individual consciousness: “Each new phase of consciousness arises within a structural context that changes the two fundamental coordinates of a social system: time and space. Each leap of civilisation that reshapes time and space has forged individual consciousness by redefining the perception of reality and experience” (p.54). In this social evolution, which Iorio traces in its various stages from agricultural societies up to the second industrial revolution, mankind has refined its sensitivity towards the other, albeit in an uneven manner, and “fewer and fewer groups are considered ‘other’” (p.63).

While Iorio looks back at socio-historical evolution, Paulo Henrique Martins looks at how care can be an alternative to the crisis of the old emancipatory perspectives based on technical, economic, cultural and political rationalities inherited from the Enlightenment; and the exhaustion of a capitalist development model incapable of responding to the complexity of the systemic potential of human action. As current modes of governance become increasingly ineffective at managing the dire threats to humanity’s destiny in the 21st century, pluriversalism and care for the world become the new utopias that can make human beings walk towards a common affective horizon.

While the first part of the volume lays the theoretical and methodological foundation of social love, the rest of the book presents a number of studies addressing its four dimensions: overabundance, care, universalism, and recognition.

Overabundance, to reiterate, is an identifier for those types of behaviour “exceeding shared expectations and doing more than what the situation requires or more than what has been received according to a given measure.” This concept may recall Mauss’ (2016) idea of *gift*. However, while the latter centres around the obligations to give, to receive, and, most importantly, to reciprocate, the former is a kind of relationship that typically lacks calculation, quantification and exchange. In other terms, it does not need any antecedent with one’s action and is not a means to an end.

Sari Hanafi shows in his theoretical contribution how he employed social love in his own sociological work as an heuristic approach to understanding the communities he studied in the Arab world, and how this approach marks his commitment to these communities. Starting from his biographical and professional trajectory – a Syrian activist who grew up in a refugee camp in Syria, “haunted by a duality: oppressive Israeli colonial practices and authoritarianism in Syria” (p.89), and was arrested several times but managed to study in France – he concludes by arguing in favour of a morally engaged sociology “that always thinks about how to dialogue with people and how to acknowledge people’s cultural heritage, in order to sensitize people to ethics and moral philosophy” (p. 97).

In their contribution Marco Palmieri and Chiara Iannaccone propose operationalizing overabundance through a global index, the World Love Index, which measures the loving attitude both of citizens using individual data and of institutions using ecological data, while Adrian Scribano and Geoffrey Pleyers focus on collective action in their chapters. The former, who has emblematically titled his chapter “Collective action and love”, shows how hundreds of collective actions, especially in Latin America, are performed based on a shared energy configured around the proximity and communality that produce and propitiate filial love. His argument is that love as an interstitial practice produces a set of collective practices that it is possible to observe in connection with the politics of sensibilities and social conflict. Geoffrey Pleyers, instead, focuses on the timely issue of social movements during the Covid 19 pandemic, one of the multiple crises that characterise our contemporaneity and challenge our living together. The author shows how social movements and mutual aid have an important role in incentivizing, fostering, and organizing solidarity and care through mutual aid initiatives. He also shows the intersections of collective action aimed at social love and collective action aimed at political transformation. The examples he provides, from feminists sheltering women victims of domestic violence to the networks of mutual aid protecting against Covid in Brazilian favelas, go well beyond social love and directly challenge political and patriarchal power.

Care, understood as a form of one self’s opening to the other and to the world, is the second key dimension of social love and has two fundamental requisites: one is the eminently practical dimension; the other is the attention and empathy that allow you to experience the other. As Cataldi and Iorio write (p. 141): “Taking care of others starts from a specific perspective: taking charge of the suffering of the other. For this reason, often it expresses itself by privileging vulnerable people, the least ones and the excluded. But people are closely interconnected with nature, so loving others also means loving future generations and overcoming production as the only form of relationship with the world.” This point is conceptualised further by Felipe Campello, who, in his theoretical chapter, proposes moving beyond an anthropocentric approach to love and extending its relational logic to include our relation to nature. Inspired by the work of Honneth (2010), he shows that love represents a prerequisite for public life and solidarity, not only between human beings, but also between human beings and nature. While the relationship between human beings and nature is at the core of the *insight* chapter, the *evidence* contributions examine how care can be articulated in contemporary welfare systems, particularly in the Mediterranean area. Andrea Gallelli, Paolo Contini and Angela Mongelli, focusing on contexts of deprivation and educational poverty in Bari, South Italy, and using the results from the World Love Index (WLI) (Palmieri et al., 2021; see also chapter three in this edited collection), show how education, one possible way of tackling human deprivation, can contribute to work on social love, and specifically to the dimension of care as a way of prioritizing benefit to others. Giuseppe Pellegrini and Licia Paglione return to the pandemic, illustrating the results of a survey on the pro-social and altruistic behaviours of young Italians in the lockdown months. Using Sorokin’s approach to altruism as a form of social love (Sorokin, 1950, 1954), the authors focus on indicators that empirically observe the first months of lockdown in the Italian context. They demonstrate how physical distancing did not necessarily implied social distancing. On the contrary, the lockdown was also a generative and regenerative form of action, as well as an expression of social love. Barbara Sena presents some preliminary reflections on social love in the health professions, recommending some possible research developments. She argues that most of the literature on love in professional practice is vague and equivocal. Therefore, there is still a lack of awareness of its specific characteristics that would make it distinct from or link it to such terms as caring, responsibility, benevolence or attention. This leaves room for more focused empirical research to better understand the function of social love in professional practices, especially in healthcare settings.

The last two chapters in this part address the issues of the welfare state in the European and South American contexts. Luigi Gui and Tiziano Vecchiato recommend a generative welfare involving people’s co-responsibility. According to the authors, the main challenge that current welfare states are facing is to ensure sustainability and distributive equity while considering the ethical dilemmas to be addressed by this difficult balance. The solution identified in this chapter is a generative welfare state that enhances the capabilities and potential of each individual, not only those who receive care but also those who provide it. For this reason, maximum generativity is achieved not only by enhancing capacities and preventing beneficiaries from becoming passive recipients of subsidies and services, but also by moving away from the procedure-based work and tasks of social workers. In their chapter, Rolando Cristao, Marcelo Salas and Clara Desalvo bring in the Latin American perspective, investigating experiences of activating social love in contexts of structural inequalities further enhanced by Covid 19. While the previous chapter suggests changes that mainly focus on forms of participation and activation of both service users and service providers, Cristao, Salas and Desalvo argue that structural reforms are needed, in both the short and long term, that move towards a universal, solidarity-based and redistributive policy, and that can only be achieved on the basis of social and fiscal pacts.

The fourth part on universalism, the recognition that social love is not only addressed to people in one’s own social circle or to similar people to oneself, but goes beyond the logic of in-group relationships, is opened by Alain Caille, the French sociologist who founded the Anti-Utilitarist Movement in the Social Sciences, inspired by the thought of Marcel Mauss and Karl Polanyi. In his short but effective chapter, Caille presents the latest developments of the convivialist manifesto. In his own words, convivialism is “a word, a signifier and a symbol. It is a symbol of hope.” It is the attempt to replace the grand narratives of hope from the last two centuries: liberalism, socialism, communism and anarchism, which have been defeated by neoliberalism in one way or another, with a new one, not based on utilitarian reason but on harmony between humans and harmony between humans and nature. How can this project of hope can be achieved? According to Caille, it could be realised through the creation of a World Citizens’ Parliament, which could embody the moral conscience of mankind at its most general and speak on behalf of the whole of humanity. To use Caille’s definition, this would be a ‘pluriversalist’ arena that transcend special interests and is made possible by new digital tools.

Fabrizio Martire and Paolo Parra Saiani discuss the dichotomy of universalism-particularism today and present the results of a statistical cross-national analysis based on two cross-continental survey projects: the Gallup World Poll and the World Values Survey. The comparison of these surveys’ findings, particularly between Europe and South America, show that, while Europe seems affected by a “not-engaged universalism”, i.e., a generally positive and not hostile attitude towards different social groups that are kept at a distance, South America is characterised by a “conditional universalism”, which is incomplete, since universalism should be unconditional.

Emanuele Polizzi concludes this part on universalism with an essay on how the institutional dimension of love and the care of common goods is challenged in current times. In his theoretically-informed essay, Polizzi connects with the debate on the commons and the nature of current welfare systems. He argues that social love is not only a face-to-face relationship between donor and recipient, but can also have an impersonal dimension and be a property of state institutions. In a way, it is precisely the disappearance of a binary relationship in favour of a relationship mediated by a third party, i.e. public institutions, that makes possible its overabundance and universalism. However, Polizzi warns, the characteristic of publicity and the effective universalism of public institutions cannot be taken for granted and in cases where they are jeopardized by forms of privatization even their universalistic character can be exposed to possible decay.

The final part of the volume addresses the fourth dimension of social love: recognition. The key idea is that social love is a bond that enhances differences rather than flattens them by allowing people to be themselves to a stranger. Overabounding always applies to people who are socially, historically, and culturally situated and irreducible to one another but, at the same time, it requires some form of mutual recognition. In this sense, social love is suitable for plural contemporary society with its emphasis on differences and identities. Recognition is also an eminently political issue, as evidenced by research on social movements (Mulholland et al., 2018), and the contributions in this part address this dimension through the lenses of political science and political sociology. André Magnelli proposes a reflection on love and recognition in socialisation processes and in the contemporary political horizon that is challenged by populism. In his essay Magnelli interrogates love as an experience central not only to the formation of individuals, couples, families, and private bonds, but also to the construction of the common good, the constitution of public spaces, and the dynamization of democratic institutions. He addresses these issues by using Pierre Rosanvallon’s critical theory of populism (2020) and argues that “the populist economy of emotions and passions generates a self- destruction of democracies” (p. 267). William Calvo Quirós, in his empirical contribution, presents case studies of love and forgiveness in US communities. He shows that forgiveness can be a revolutionary and transformative act that emerges as an interpellation to invoke a new method for long-lasting racial relationships. Cultural recognition is also the focus of the contribution on current events written by three authors specialising in pedagogy, anthropology and intercultural sociology, William Calvo Quirós, Agnès Marie Kehuo and Antonio Mendes da Costa Braga. Using a Southern decolonial perspective and starting from their experiences as scholars from the South, the authors present examples of how love takes shape in the practices of different cultures. Their aim is to explore crucial questions around the tensions created between the discourses of love and coloniality and emphasize the universality of love as an expression of what it means to be human, but also “as a subversive typology for change” (p.311).

## Conclusion

In conclusion, this is a highly interesting and topical collection of essays on love and its social implications. It fills a gap in the sociological debate, and also represents a step forward in research on this topic. The two editors, Cataldi and Iorio, were able to bring together scholars from different disciplines and provide a coherent reading of social love and its practical implications on contemporary problems. They have convincingly documented how love, far from being just a private and personal emotion, is political in that it involves several dimensions of public life, from the welfare state and the redistribution of resources to the recognition of diversity.

At this point, I would like to suggest a couple of possible developments in the research. First, it is important to broaden the approach beyond Europe and Latin America to see how the four dimensions investigated in this volume are articulated on other continents. While much of the focus is on those two continents, it would be interesting to have a more global perspective and investigate what is going on in other continents characterised by different cultures, political institutions, issues and standard of living. Second, another area that is present in the volume, but worthy of further investigation, is that of collective action. Collective movements, including workers’ and women’s movements, environmentalists, solidarity actions in favour of migrants, etc., are not only characterised by the use of contentious repertoires, but can also be seen as driven by social love: towards oppressed people, the environment, and human beings in general (Montagna, 2007a; 2007b). The action of activists in social movements is often *overabounding*, it is guided by forms of altruistic *care*, some of the principles that move them are *universalistic* while aiming at mutual *recognition*. A possible line of research, therefore, might aim to investigate how these movements articulate the four dimensions of social love and how their action is informed by them.

Finally, while most of the contributions in this volume have mostly touched on welfare and the recent Covid-19 emergency, it would be interesting to see how different actors articulate the theme of social love in other contexts of crisis, such as armed conflicts and those produced by climate change. These are just some of the areas that scholars of social love might want to develop further. Their indication, however, does not diminish the value of this volume.

## Data Availability

Not applicable.

## Footnotes

1 On Pitirim Sorokin see Cataldi’s, and Pellegrini’s and Paglione’s chapters in the volume.

2 For a more in-depth overview of the reflections on love, I recommend reading Silvia Cataldi’s excellent chapter in the first part of the volume *Imagining on the shoulders of giants* (Routledge, 2022).
